# Association study of polymorphisms in the *ABO* gene with ischemic stroke in the Chinese population

**DOI:** 10.1186/s12883-016-0671-7

**Published:** 2016-08-19

**Authors:** Xiaoming Ling, Yansong Zheng, Jing Tao, Zhezhou Zheng, Lidian Chen

**Affiliations:** 1 Fuzhou General Hospital of Nanjing Military Command, Fuzhou, 350025 China; 2 The Health Management Institute of the General Hospital of PLA, Beijing, 100853 China; 3 College of Rehabilitation Medicine, Fujian University of Traditional Chinese Medicine, Fuzhou, 350122 China; 4 Fujian Key Laboratory of Exercise Rehabilitation, Fuzhou, 350003 China; 5 The Second People’s Hospital Affiliated to Fujian University of Traditional Chinese Medicine, Fuzhou, 350122 China; 6 Fujian University of Traditional Chinese Medicine, Fuzhou, 350122 China

**Keywords:** Ischemic stroke, ABO, Single nucleotide polymorphism, Association study

## Abstract

**Background:**

Ischemic stroke is the main cause of mortality and disability in older people worldwide. Recently epidemiological studies indicate that ischemic stroke is a complex disorder with a strong genetic component. Genome-wide association studies (GWAS) identified several single nucleotide polymorphisms (SNPs) associated with coronary artery disease (CAD) and myocardial infarction (MI) locus in ABO gene. Our study examined the association between four variants in the *ABO* gene and the risk of ischemic stroke and its subtypes, large-artery atherosclerosis (LAA) and small-vessel diseases (SVD) in the Chinese population.

**Methods:**

In this case–control study, we recruited 1897 subjects, including 979 healthy controls and 918 ischemic stroke patients (465 with LAA and 453 with SVD). We selected four single nucleotide polymorphisms (rs579459, rs651007, rs514659 and rs529565) of the *ABO* gene and performed genotyping assays to assess the association with ischemic stroke and its subtypes.

**Results:**

We found three polymorphisms, rs579459 and rs651007 were significantly associated with LAA using additive model and rs529565 was significantly associated with LAA using additive and dominant models. And we failed to find any significant association between these SNPs and ischemic stroke and SVD in the Chinese population. However, after the Bonferroni correction for multiple comparisons, the *P*-values of these SNPs failed to exceed significant threshold under any models.

**Conclusion:**

Our findings indicated that genetic variations of *ABO* gene may contribute to susceptibility of LAA but not ischemic stroke and SVD in the Chinese population. Our preliminary results should be further validated in prospective independent studies with expanded sample size.

## Background

Stroke is one of the main causes of death and adult disability around the world [1, 2]. There are two major categories of stroke: ischemic stroke and hemorrhagic stroke, and ischemic stroke constitutes over 80 % of total stroke in origin [3]. Increasing evidence indicated that ischemic stroke is a complex clinical syndrome resulting from environmental and genetic factors [4, 5]. According to the modified Trial of Org10172 in Acute Stroke Treatment (TOAST) classification,ischemic stroke itself can be divided into five subtypes, LAA and SVD are two common etiologic subtypes of ischemic stroke [6, 7]. Conventional risk factors such as hypertension, diabetes mellitus, dyslipidemia and smoking could not completely explain all ischemic stroke risk, family and twin-based studies demonstrated that genetic factors also play a key role in the development of ischemic stroke [8, 9].

The *ABO* gene, located around 9q34.2, encodes glycosyltransferases, which catalyze the transfer to different carbohydrate groups onto the H antigen, thus forming A and B antigens of the ABO system [10, 11]. In recent years, several studies have found that genetic variants of *ABO* gene were associated with several diseases. Previous studies have identified rs579459 in *ABO* as genetic variants associated with the risk of CAD in European descent [12]. Recent study also identified the *ABO* rs579459 polymorphism as genetic variants significantly associated with venous thromboembolism (VTE) [13]. However, fewer studies focus on the association between *ABO* gene and ischemic stroke [14–16]. Ischemic stroke and CAD have several common risk factors and genetic susceptibility, especially for LAA and CAD. Taking all these considerations together, the *ABO* gene may be a promising candidate gene of ischemic stroke. Recent GWAS showed that three SNPs (rs505922, rs643434, and rs651007) of *ABO* gene were associated with ischemic stroke and its subtypes in the European population [15]. However, there were no independent replication studies regarding the association between *ABO* gene and ischemic stroke in Chinese population.

Against this background, in the present study we aimed to investigate the association between four SNPs (rs579459, rs651007, rs514659 and rs529565) of *ABO* gene and ischemic stroke susceptibility in the Chinese population.

## Methods

### Subjects

Our study sample recruited 979 healthy controls and 918 ischemic stroke patients, including 465 with LAA and 453 with SVD who presented consecutively to the Second People’s Hospital Affiliated to Fujian University of Traditional Chinese Medicine, Fujian Provincial Hospital, Fuzhou General Hospital of Nanjing Military Command and Fujian University of Traditional Chinese Medicine Subsidiary Rehabilitation Hospital during August 2013 to December 2014. The Second People’s Hospital Affiliated to Fujian University of Traditional Chinese Medicine as a leader of the organization jointed three other hospitals to collect the object of study. All case and control subjects were unrelated to one another and were recruited from the Chinese population. Clinical diagnoses of ischemic stroke were confirmed through computed tomography (CT) and/or magnetic resonance imaging (MRI) scans of the brain. The brain images were independently assessed by two well-trained technologist and physician. The common subtypes (LAA and SVD) of ischemic stroke were determined by the modified TOAST classification system [6]. Control subjects were recruited from the health management of the Second People’s Hospital Affiliated to Fujian University of Traditional Chinese Medicine. Controls with stroke and other neurological diseases and cardiovascular diseases were excluded in this study. The questionnaire was designed to collect demographic characteristics, clinical vascular variables, and medical histories for both cases and controls. Conventional vascular risk factors including hypertension, diabetes mellitus, and dyslipidemia were evaluated through WHO/ISH criteria. This study was approved by the institutional review boards of all participating hospitals. Written informed consent and peripheral blood samples were obtained from patients and controls before they attended our study.

### SNP selection and genotyping

We selected four SNPs of the ABO gene, including rs579459, rs651007, rs643434, and rs505922. For two SNPs, rs643434 and rs505922 failed assay primer design, alternative SNPs in complete Linkage disequilibrium (LD) were chosen. By HapMap, rs643434 and rs505922 were replaced respectively with rs514659 and rs529565 as in strong LD (r^2^ = 1) in the Chinese population. The SNPs were genotyped using Sequenom MassARRAY platform (San Diego, U.S) at CapitalBio Corporation (Beijing, China) and the genotyping analysis was undertaken according to the manufacturer’s protocol.

Genomic DNA was isolated from human peripheral blood samples of each individual through Wizard® Genomic DNA Purification Kit (Promega, Madison, WI, USA). DNA concentration was determined by DNA spectrophotometer (ND-1000, NanoDrop, Wilmington USA). Specific assays including a locus-specific PCR reaction based on a locus-specific primer extension reaction were designed using the MassARRAY Assay Design software package (v3.1). Mass determination was carried out with the MALDI-TOF mass spectrometer and Mass ARRAY Type 4.0 software was used for data acquisition.

### Data analysis

Association analysis was performed with PLINK software using additive, dominant, recessive and genotype models [17]. Hardy-Weinberg equilibrium (HWE) were performed for each SNP. Logistic regression was used for risk stratification with or without covariate adjustments determined by significant differences between ischemic stroke patients and controls, such as age, gender, hypertension, and diabetes mellitus.

## Results

### Clinical characteristics of total ischemic stroke patients and controls

The demographic characteristics and clinical vascular variables for the 918 ischemic stroke patients (51 % LAA and 49 % SVD) and 979 healthy control subjects used in this study are shown in Table 1. Distribution of age and gender between cases and controls were significantly different. Other risk factors such as smoking, drinking, and diabetes mellitus were found to be more prevalent in cases compared to controls. Hypertension was significantly lower in ischemic stroke cases than in controls. There were no significant differences in levels of triglyceride between the ischemic stroke cases and the controls, but total cholesterol, low density lipoprotein, and high density lipoprotein levels were significantly lower in ischemic stroke cases compared to controls.

**Table 1:** Demographic information of participants

Group	Cases (*n* = 918)	Controls (*n* = 979)
Large Vessel Disease, *n* (%)	465 (51 %)	/
Small Vessel Disease, *n* (%)	453 (49 %)	
Age, years	69.39 ± 10.45*	67.04 ± 10.26
Male, *n* (%)	601 (65 %)*	562 (57 %)
Female, *n* (%)	317 (35 %)*	417 (43 %)
Hypertension, *n* (%)	216 (24 %)*	316 (32 %)
Diabetes, *n* (%)	598 (65 %)*	126 (13 %)
Smoking, *n* (%)	626 (68 %)*	170 (17 %)
Drinking, *n* (%)	736 (80 %)*	81 (8 %)
Triglyceride, mmol/L	1.59 ± 0.94	1.60 ± 0.91
Total Cholesterol, mmol/L	4.62 ± 1.21*	5.22 ± 1.08
Low Density Lipoprotein, mmol/L	3.04 ± 1.66*	3.42 ± 1.00
High Density Lipoprotein, mmol/L	1.20 ± 0.52*	1.35 ± 0.34

Data were shown as mean ± standard deviation (SD) or as *n* (%). Significant differences between cases and controls were indicated with an asterisk (*)

### Comparison of allele and genotype frequencies in ischemic stroke

The association between the four SNPs in *ABO* gene and the risk of ischemic stroke was analyzed using additive, dominant, genotype, and recessive models. However, the polymorphism rs514659 was not found to be in HWE in control subjects, therefore excluded from further statistical analyses. The observed allele and genotype frequencies for ischemic stroke cases and controls are shown in Table 2, rs579459 was significantly associated with the risk of ischemic stroke using dominant model (*p* = 0.04, OR = 1.22, 95 % CI = 1.00-1.48). The allele frequencies of the other two SNPs (rs651007 and rs529565) showed no difference between the ischemic stroke and the control group (*p* > 0.5). However, the association between rs579459 and ischemic stroke failed to remain significance after logistic regression analysis adjusting for age, gender, hypertension, diabetes mellitus, dyslipidemia, smoking and drinking status.

**Table 2:** Association between SNPs and ischemic stroke using the additive, genotype, dominant, and the recessive models

SNP	Model	Allele or geno	case	control	Unadjusted OR (95 % CI)	Unadjusted *p*-value	Adjusted OR (95 % CI)	Adjusted *p*-value
rs579459	Additive	**C**/T	363/1455	334/1576	1.18 (1.00-1.39)	0.05	1.18 (0.97-1.43)	0.10
	Dominant	CC + CT/TT	326/583	300/655	1.22 (1.00-1.48)	**0.04**	1.20 (0.95-1.52)	0.12
	Recessive	CC/CT + TT	37/872	34/921	1.15 (0.71-1.85)	0.57	1.31 (0.74-2.31)	0.36
rs651007	Additive	**T**/C	349/1421	337/1575	1.15 (0.97-1.36)	0.10	1.15 (0.94-1.40)	0.17
	Dominant	TT + CT/CC	314/571	304/652	1.18 (0.97-1.43)	0.09	1.16 (0.92-1.46)	0.22
	Recessive	TT/CT + CC	35/850	33/923	1.15 (0.71-1.87)	0.57	1.35 (0.76-2.40)	0.31
rs529565	Additive	**C**/T	679/1087	709/1201	1.06 (0.93-1.21)	0.41	1.06 (0.90-1.24)	0.48
	Dominant	CC + CT/TT	540/343	565/390	1.09 (0.90-1.31)	0.38	1.08 (0.86-1.35)	0.52
	Recessive	CC/CT + TT	139/744	144/811	1.05 (0.82-1.36)	0.69	1.08 (0.79-1.47)	0.62

All SNPs were analyzed under additive, genotype, dominant (Dom) and recessive (Rec) models; OR: odds ratio; CI: confidence interval; unadjusted *P*-value from *t*-test; adjusted *P*-value using logistic regression analysis with age, gender, hypertension, diabetes, and dyslipidemia as covariates. Significant *P* values (*p* < 0.05) are in bold and p* < 0.017 (Bonferroni multiple correction threshold)

### Comparison of allele and genotype frequencies in LAA

As shown in Table 3, we also observed the association between the three SNPs of *ABO* gene and LAA occurrence under additive, dominant, genotype, and recessive models. Three polymorphisms, rs579459, rs651007, and rs529565 were significantly associated with LAA in both additive and dominant models. After adjusting for age, gender, hypertension, diabetes mellitus, and dyslipidemia by logistic regression analysis, two polymorphisms, rs579459 and rs651007 were remained significantly association with LAA using additive model and rs529565 was significantly associated with LAA using additive and dominant models. However, all *P*-values failed to reach significance after the Bonferroni adjustment for multiple comparisons.

**Table 3:** Association between SNPs and LAA using the additive, genotype, dominant, and the recessive models

SNP	Model	Allele or geno	case	control	Unadjusted OR (95 % CI)	Unadjusted *p*-value	Adjusted OR (95 % CI)	Adjusted *p*-value
rs579459	Additive	**C**/T	197/721	334/1576	1.29 (1.06-1.57)	**0.01**	1.27 (1.00-1.61)	**0.047**
	Dominant	CC + CT/TT	174/285	300/655	1.33 (1.06-1.68)	**0.015**	1.28 (0.96-1.70)	0.09
	Recessive	CC/CT + TT	23/436	34/921	1.43 (0.83-2.46)	0.19	1.70 (0.88-3.29)	0.11
rs651007	Additive	**T**/C	193/703	337/1575	1.28 (1.05-1.56)	**0.01**	1.30 (1.02-1.64)	**0.033**
	Dominant	TT + CT/CC	170/278	304/652	1.31 (1.04-1.66)	**0.02**	1.29 (0.97-1.71)	0.08
	Recessive	TT/CT + CC	23/425	33/923	1.51 (0.88-2.61)	0.13	1.89 (0.97-3.65)	0.06
rs529565	Additive	**C**/T	374/518	709/1201	1.22 (1.04-1.44)	**0.01**	1.24 (1.02-1.51)	**0.03**
	Dominant	CC + CT/TT	295/151	565/390	1.35 (1.07-1.71)	**0.01**	1.39 (1.05-1.84)	**0.02**
	Recessive	CC/CT + TT	79/367	144/811	1.21 (0.90-1.64)	0.21	1.24 (0.86-1.79)	0.25

All SNPs were analyzed under additive, genotype, dominant (Dom) and recessive (Rec) models; OR: odds ratio; CI: confidence interval; unadjusted *P*-value from *t*-test; adjusted *P*-value using logistic regression analysis with age, gender, hypertension, diabetes, and dyslipidemia as covariates. Significant *P* values (*p* < 0.05) are in bold and p* < 0.017 (Bonferroni multiple correction threshold)

### Comparison of allele and genotype frequencies in SVD

To explore whether the *ABO* polymorphisms are confined to a specific subtype, we also evaluated the association between the three SNPs of *ABO* gene and the risk of SVD. As shown in Table 4, no significant associations were observed for the allele and genotype frequencies between the cases and controls in all three SNPs.

**Table 4:** Association between SNPs and SVD using the additive, genotype, dominant, and the recessive models

SNP	Model	Allele or geno	case	control	Unadjusted OR (95 % CI)	Unadjusted *p*-value	Adjusted OR (95 % CI)	Adjusted *p*-value
rs579459	Additive	**C**/T	166/734	334/1576	1.07 (0.87-1.31)	0.54	1.08 (0.85-1.39)	0.53
	Dominant	CC + CT/TT	152/298	300/655	1.11 (0.88-1.41)	0.38	1.10 (0.83-1.47)	0.51
	Recessive	CC/CT + TT	14/436	34/921	0.87 (0.46-1.64)	0.67	1.09 (0.51-2.32)	0.83
rs651007	Additive	**T**/C	156/718	337/1575	1.02 (0.82-1.25)	0.89	1.03 (0.80-1.33)	0.80
	Dominant	TT + CT/CC	144/293	304/652	1.05 (0.83-1.34)	0.67	1.04 (0.78-1.39)	0.81
	Recessive	TT/CT + CC	12/425	33/923	0.79 (0.40-1.54)	0.49	1.05 (0.47-2.32)	0.91
rs529565	Additive	**C**/T	305/569	709/1201	0.91 (0.77-1.07)	0.26	0.94 (0.77-1.14)	0.51
	Dominant	CC + CT/TT	245/192	565/390	0.88 (0.70-1.11)	0.28	0.87 (0.66-1.15)	0.33
	Recessive	CC/CT + TT	60/377	144/811	0.90 (0.65-1.24)	0.51	1.01 (0.69-1.50)	0.95

All SNPs were analyzed under additive, genotype, dominant (Dom) and recessive (Rec) models; OR: odds ratio; CI: confidence interval; unadjusted *P*-value from *t*-test; adjusted *P*-value using logistic regression analysis with age, gender, hypertension, diabetes, and dyslipidemia as covariates. Significant *P* values (*p* < 0.05) are in bold and p* < 0.017 (Bonferroni multiple correction threshold)

## Discussion

The *ABO* gene is located near the end of the long arm of chromosome 9 and encodes glycosyltransferases, which add sugar residues to the H-antigen producing A or B antigens of the ABO system. Previous studies found that the non-O phenotypes were more frequent in ischemic stroke patients than controls and was associated with an increased risk of MI and CAD [18–20]. In consistent with these results, previously study showed that compared with the O phenotype, non-O phenotypes associated with an increased risk of stroke [21]. In contrast, other study did not detect significant association between ABO blood group and ischemic stroke and any of the four main etiologic subtypes of ischemic stroke [14]. Ischemic stroke is a complex disease with different pathophysiology and risk factors. It is important to investigate risk factors in different etiologic subtypes. Some subtypes of ischemic stroke and CAD shared many common risk factors, for example, atherosclerosis plaque were observed in both LAA and CAD as a common pathophysiologic mechanism. Consequently, it was speculated that genetic variants of *ABO* gene associated with CAD [12], may be also associated with LAA.

Previous studies have found that polymorphisms of *ABO* gene were associated with many diseases [12, 13, 15]. As reported previously that rs579459 in *ABO* gene was associated with CAD in Caucasians [12]. Fewer studies have investigated the genetic association between *ABO* polymorphisms and ischemic stroke. Early studies reported that *ABO* gene variants (rs651007, rs643434, and rs505922) are associated with LAA and cardioembolic stroke in the European population [15]. However, there was no study reported the association between *ABO* gene and ischemic stroke in the Chinese population. Hence, in our case–control study, we investigated the association of four polymorphisms in *ABO* gene with the risk of ischemic stroke and its main subtypes. Our results supported previous observations that ischemic stroke in particular LAA and CAD share several risk factors. We found that rs579459 in *ABO* gene was associated with LAA. In line with previous study [15], our current study found significant associations between *ABO* SNPs (rs651007 and rs529565) and LAA, and these SNPs were failed to be associated with ischemic stroke and SVD, although the association disappeared after the Bonferroni adjustment, which was known to be one of the most stringent methods for multiple comparisons. The observed minimal differences between the results from our results and previous study may partly due to differences in genetic background and our study have a small sample size.

Our study had a number of limitations. First, we selected four SNPs of the *ABO* gene which showed association with CAD or ischemic stroke in the European population. These SNPs merely represented limited genetic variability of *ABO* gene. The future studies will be required to confirm the association between *ABO* gene and ischemic stroke and its subtypes by high density genotyping on SNPs of *ABO* gene. Second, limited size of the cohort might reduce the power to detect association. Thus, prospective independent studies with a comparatively larger sample size are required for validation in the Chinese population.

## Conclusions

In the present study we aimed to investigate the association between ABO gene and ischemic stroke and its main subtypes in the Chinese population. We found that three SNPs (rs651007, rs579459 and rs529565) of ABO gene were significantly associated with LAA in the Chinese population, though not survived Bonferroni correction for multiple comparisons. Therefore, prospective studies with a comparatively large sample size are required to confirm the association between ABO gene and ischemic stroke in the Chinese population and to characterize the functional role of ABO underlying ischemic stroke or LAA.

## Abbreviations

SNP: Single nucleotide polymorphism
GWAS: Genome-wide association studies
CAD: Coronary artery disease
MI: Myocardial infarction
LAA: Large-artery atherosclerosis
SVD: Small-vessel diseases
VTE: Venous thromboembolism
CT: Computed tomography
MRI: Magnetic resonance imaging
LD: Linkage disequilibrium
HWE: Hardy-Weinberg equilibrium tests
OR: Odds ratio
CI: Confidence interval
